# The relationship between weight stigma and physical activity avoidance among college students: a chain mediation of body image and psychological resilience

**DOI:** 10.3389/fpsyg.2026.1757904

**Published:** 2026-04-02

**Authors:** Chen Sun, Paramasivam Muthusamy, Jiao Li, Heng Xu, Junying Du, Kaiyuan Liu, Song Wei, Lulu Zhang

**Affiliations:** 1 College of Physical Education and Health Science, Yibin University, Yibin, China; 2 Faculty of Education and Liberal Arts, INTI International University, Nilai, Malaysia; 3 Department of Education, Graduate School, Kookmin University, Seoul, Republic of Korea

**Keywords:** body image, chain mediation, physical activity avoidance, psychological resilience, weight stigma

## Abstract

**Background:**

Physical activity avoidance among college students is a public health issue related to their health and well-being. Factors such as weight stigma, body image, and psychological resilience are associated with physical activity avoidance behaviors and may interact in complex ways.

**Purpose:**

With an emphasis on the mediating roles of body image and psychological resilience, this study, which is based on stress-coping theory, attempts to investigate the relationship between weight stigma and college students’ physical activity avoidance.

**Methods:**

A random sampling approach collected 576 valid responses from various universities (47.40% male, 52.60% female). The Weight Self-Stigma Scale, Body Image State Scale, Connor-Davidson Psychological Resilience Scale, and Physical Activity Avoidance Tendency Scale were used for measurement. Data were analyzed using the chain mediation model (Model 6) in PROCESS 4.0 within SPSS 26.0.

**Results:**

Physical activity avoidance was positively correlated with weight stigma (*β* = 0.255, *p* < 0.001). Resilience and body image had a significant indirect effect as well [*β* = 0.024, 95% CI (0.012, 0.039)], making up 6.43% of the overall effect.

**Conclusion:**

Weight stigma relates to physical activity avoidance among college students, mediated by body image and resilience. The results identify cognitive and emotional pathways associated with social stressors and health behaviors among college students, offering a theoretical basis and intervention insights for promoting good health and well-being within university populations.

## 1 Introduction

The lack of physical activity among college students is a significant global health concern, with links to both mental and physical health risks such as depression and anxiety (She et al., 2023). Physical activity avoidance, a critical factor in understanding sedentary behaviors, plays a key role in addressing these risks (Huang et al., 2025). According to the World Health Organization, 27% of adults and more than 80% of adolescents do not engage in the recommended amounts of physical activity (Yi et al., 2024). Among college students, the prevalence of physical inactivity is widespread, with rates as high as 65% in Australia and 41% among undergraduates (Johannes et al., 2022). In China, physical inactivity rates are similarly concerning, ranging from 40 to 50% (Peng et al., 2022). These statistics highlight the pressing need to explore the psychological factors contributing to physical activity avoidance among college students.

The development of physical activity avoidance is influenced by both external social factors and internal psychological processes. External factors, such as weight stigma, peer ridicule, and unwelcoming sports environments, significantly contribute to this behavior (Huang et al., 2025; Li et al., 2025). Weight stigma, as a common social stressor, involves prejudice, discrimination, and negative stereotypes based on an individual’s weight or body shape. These social judgments often increase social anxiety and discomfort in physical activity settings, directly contributing to physical activity avoidance (Ajibewa et al., 2024). In addition to societal constraints, poor psychological resilience, low self-esteem, and a negative body image are important factors in the development of physical exercise avoidance (Greenleaf and Hauff, 2024; Soraci et al., 2024). Avoiding physical activity can be directly influenced by one’s body image, or how they view and assess their body, especially when weight stigma is present (Czepczor-Bernat, 2022). Furthermore, psychological resilience—defined as the capacity to bounce back from stress—is crucial for controlling the detrimental effects of outside stresses and reducing the dangers of sedentary behavior (Luo et al., 2025).

Previous studies have explored the relationships between weight stigma, body image, psychological resilience, and physical activity avoidance (Dever et al., 2025; Saffari et al., 2025; Voon et al., 2022), but the continuous interaction of these factors remains underexplored. Although stress-coping theory (Folkman, 1984) has been widely applied to acute stressors, this study extends its application to chronic social stressors, such as weight stigma, to offer a more nuanced understanding of how these stressors influence college students’ physical activity avoidance. According to the stress-coping framework, stressors are evaluated in two stages: primary appraisal and secondary appraisal. In the primary appraisal phase, weight stigma is viewed as a threat to self-image and body worth, which is associated with emotional distress, particularly body image dissatisfaction (He et al., 2025). This stage reflects how weight stigma is internalized, with individuals perceiving their bodies negatively due to societal standards and judgment. Body image dissatisfaction thus represents the initial cognitive evaluation, where individuals assess their body as inadequate or flawed. This unfavorable body image affects a person’s psychological resilience, or their capacity to handle stress and hardship, throughout the secondary assessment stage (Luo et al., 2025). In particular, psychological resources can be depleted by the anguish associated with negative body image, which lowers a person’s confidence in their capacity to handle social pressures. This reduction in resilience can make it more difficult to handle the emotional strain of weight stigma, which may lead to increased physical activity avoidance as a coping response (Dursun et al., 2024). Thus, body image serves as the primary appraisal, where individuals assess their body’s adequacy, and psychological resilience functions as the secondary appraisal, shaping their ability to adapt to and manage stress related to weight stigma.

**Figure 1:**
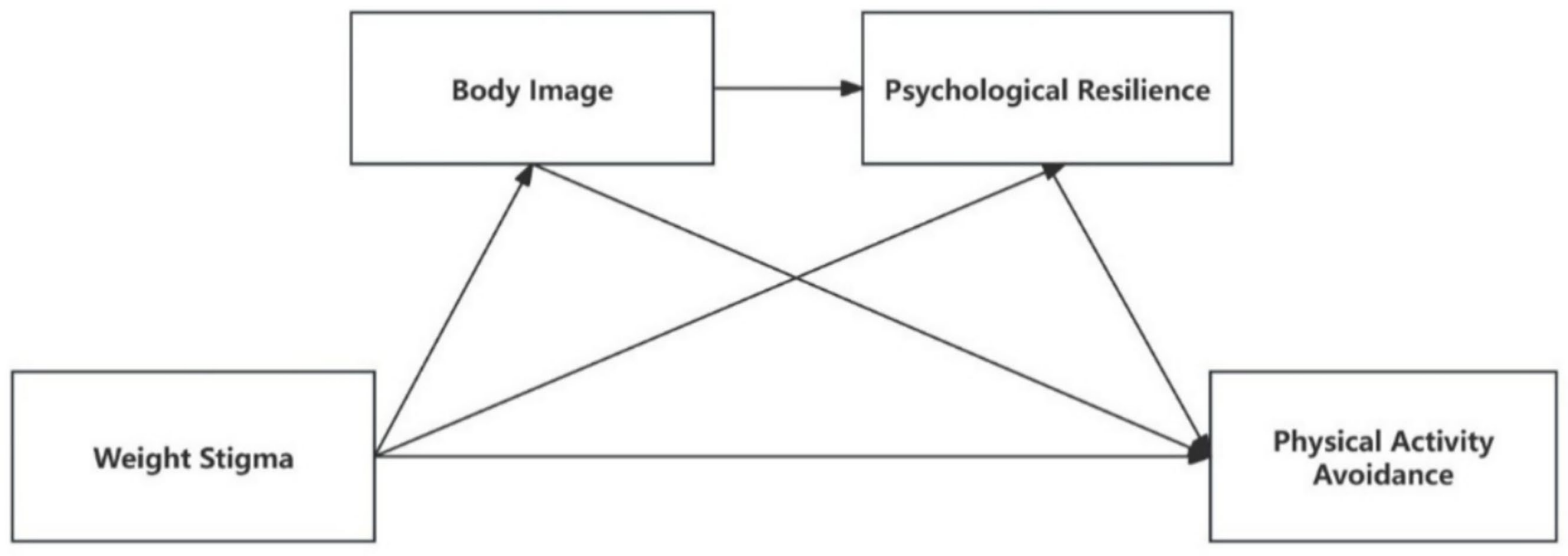
Theoretical model of research.

By integrating body image and psychological resilience into the stress-coping framework, this study enhances our understanding of how weight stigma leads to physical activity avoidance. It extends stress-coping theory by showing how chronic stressors like weight stigma are mediated by these psychological processes. This research provides a clearer explanation of how weight stigma impacts physical activity and offers a framework for developing interventions to promote physical activity among college students. The following hypotheses are put forth in this study based on this framework (see Figure 1):

H1: Weight stigma is positively associated with physical activity avoidance.

H2: Body image mediates the association between weight stigma and physical activity avoidance.

H3: Psychological resilience mediates the association between weight stigma and physical activity avoidance.

H4: Body image and psychological resilience sequentially mediate the association between weight stigma and physical activity avoidance.

## 2 Methods

### 2.1 Sample and data collection procedure

Data were obtained from September to October 2025 using an online survey platform (https://www.wjx.cn/) from four comprehensive universities located in mainland China. These universities included two public universities and two private universities, with an approximate total enrollment of 68,000 undergraduate students, providing a diverse institutional context. The sampling frame consisted of full-time undergraduate students enrolled at these universities during the 2025 fall semester. After obtaining institutional approval, the research team accessed class-level student enrollment lists provided by academic offices. Within each participating university, classes were first stratified by academic year and discipline to ensure representation across grades and fields of study. To ensure that every student had an equal chance of being requested to participate, students were then chosen at random within each class using a computerized random number generator. Course instructors informed selected students about the study during regular class sessions, emphasizing voluntary participation, anonymity, and confidentiality. The survey link was distributed only to the randomly selected students, and each participant was assigned a unique identification code to prevent duplicate responses and to maintain data integrity. No incentives were provided.

According to Kline (2018), who recommends a minimum ratio of 10 participants per questionnaire item, the 44-item survey required at least 440 participants. Allowing for an estimated 20% attrition rate, the target sample size was set at 528. A total of 576 valid surveys were collected from the 650 students who were asked to participate, yielding an 88.62% response rate. 74 questionnaires were excluded due to missing data or patterned responding.

Lastly, it should be mentioned that the cross-sectional design used in this work restricts the ability to draw conclusions about the suggested mediation pathways. Although the hypothesized relationships are grounded in stress-coping theory, the mediation analyses reflect associational patterns rather than temporal or causal processes, and the findings should be interpreted accordingly.

The demographic distribution of the 576 participants is displayed in Table 1. Although data were collected from four universities, the survey questionnaire did not include university-specific identifiers, as the primary focus was on overall participant characteristics rather than institutional comparisons. Therefore, we report overall frequencies and percentages for all demographic variables. Among the participants, 273 were men (47.4%) and 303 were women (52.6%). Most were aged 18–20 years (40.5%), with a relatively even distribution across grades. Social sciences represented the largest group (42.5%). Using World Health Organization (WHO) standards, 71.4% of the participants were categorized as normal weight.

**Table 1:** Participant demographic characteristics.

Variable	Option	Frequency	Percentage (%)
Gender	Male	273	47.4
	Female	303	52.6
Age	18–20 years	233	40.5
	20–22 years	197	34.2
	Over 22 years	146	25.3
Grade	Freshman	163	28.3
	Sophomore	173	30.0
	Junior	128	22.3
	Senior	112	19.4
Major	Humanities	176	30.6
	Social Sciences	245	42.5
	Natural Sciences	155	26.9
BMI (classification)	Underweight (BMI < 18.5)	65	11.3
	Normal (BMI 18.5–24.9)	411	71.3
	Overweight (BMI 25.0–29.9)	100	17.4
BMI (continuous)	M ± SD	23.1 ± 3.2	

BMI, body mass index; M, mean; SD, standard deviation.

### 2.2 Measurement tools

#### 2.2.1 Weight stigma

We used the 12-item Weight Self-Stigma Questionnaire (WSSQ), developed by Lillis et al. (2010), to gauge stigma fear and self-deprecation. A 5-point Likert scale was used to grade the responses; higher scores indicated greater weight self-stigma. In Chinese samples, the scale has demonstrated good dependability (Fu et al., 2025). In this study, Cronbach’s *α* was 0.916, reflecting excellent internal consistency.

#### 2.2.2 Body image

This study used the Body Image State Scale (BISS), developed by Cash (2002) and adapted by Wang et al. (2022). The scale consists of six items rated on a 9-point scale. In this study, Cronbach’s α was 0.867, indicating good internal consistency.

#### 2.2.3 Psychological resilience

The Connor-Davidson Resilience Scale, developed by Campbell-Sills and Stein (2007), was used. Cheng et al. (2020) offered the Chinese adaption, which consists of 10 items with a 5-point Likert scale for rating. In the present study, Cronbach’s *α* was 0.909, indicating excellent reliability.

#### 2.2.4 Physical activity avoidance

The Tendencies and exercise Participation Scale (TAPAS), created by Bevan et al. (2022), was used to measure physical exercise avoidance. Ten items on a 5-point Likert scale are included in the scale. For instance, “When I participate in sports, I worry that others might notice my body flaws.” Prior studies have confirmed the scale’s validity and reliability in Chinese samples (Huang et al., 2025). In the present study, Cronbach’s α reached 0.896, indicating good internal consistency.

## 3 Statistical analysis

AMOS 26.0 and SPSS 26.0 were used to examine the data. First, concept validity and model fit were evaluated using confirmatory factor analysis. Pearson correlations and descriptive statistics were then computed. Harman’s single-factor test and the unmeasured latent method construct (ULMC) technique were used to investigate common method bias. Tolerance and VIF values were used to evaluate multicollinearity. Lastly, 5,000 bootstrap samples were used for mediation analysis using the PROCESS macro (Model 6) to produce 95% confidence intervals (Preacher and Hayes, 2008). If the confidence intervals did not include 0, the effects were deemed significant (Hayes and Rockwood, 2017).

## 4 Results

### 4.1 Confirmatory factor analysis

Table 2 demonstrates that all fit indices satisfied the suggested requirements, indicating a satisfactory model fit.

**Table 2:** Model fit indices.

Fit indices	Reference value	Final model
χ^2^/df	<5	1.171
RMSEA	<0.05	0.017
GFI	>0.9	0.936
AGFI	>0.9	0.928
CFI	>0.9	0.988
IFI	>0.9	0.989
TLI	>0.9	0.988

*N* = 576. χ^2^/df, Chi-square value divided by degrees of freedom; RMSEA, root mean square error of approximation; GFI, goodness-of-fit index; NFI, normed fit index; CFI, comparative fit index; IFI, incremental fit index; TLI, Tucker- Lewis’s index.

### 4.2 Composite reliability and validity

We evaluated internal consistency with composite reliability and Cronbach’s *α* (see Table 3). According to Hair et al. (2021), the composite reliability (CR) values range from 0.914 to 0.929, all exceeding the acceptable threshold of 0.70. Cronbach’s α values above 0.70 meet accepted standards (Leguina, 2015). All scales met this standard, indicating good internal consistency. We assessed convergent validity with average variance extracted (AVE), requiring values above 0.50 (Leguina, 2015), and all constructs satisfied this criterion. The Heterotrait-Monotrait ratio (HTMT) and Fornell-Larcker criteria were both used to evaluate discriminant validity. All HTMT values (range from 0.353 to 0.513) were below the conservative threshold of 0.85, as indicated in Table 4, suggesting strong discriminant validity (Henseler et al., 2015). Furthermore, Table 5 demonstrates that each construct’s square root of AVE (0.721–0.774) exceeded its correlations with other constructs, meeting the Fornell-Larcker criterion (Fornell and Larcker, 1981).

**Table 3:** Reliability and validity.

Constructs	Items	Loadings	CR	Cronbach’s α	AVE
WS	WS 1	0.707	0.929	0.916	0.520
	WS 2	0.719			
	WS 3	0.716			
	WS 4	0.725			
	WS 5	0.707			
	WS 6	0.725			
	WS 7	0.715			
	WS 8	0.725			
	WS 9	0.726			
	WS 10	0.729			
	WS 11	0.709			
	WS 12	0.751			
BI	BI 1	0.772	0.900	0.867	0.600
	BI 2	0.780			
	BI 3	0.762			
	BI 4	0.758			
	BI 5	0.799			
	BI 6	0.775			
PR	PR 1	0.730	0.925	0.909	0.551
	PR 2	0.740			
	PR 3	0.756			
	PR 4	0.778			
	PR 5	0.719			
	PR 6	0.701			
	PR 7	0.749			
	PR 8	0.730			
	PR 9	0.772			
	PR 10	0.746			
PAA	PAA 1	0.728	0.914	0.896	0.517
	PAA 2	0.725			
	PAA 3	0.703			
	PAA 4	0.702			
	PAA 5	0.755			
	PAA 6	0.711			
	PAA 7	0.716			
	PAA 8	0.712			
	PAA 9	0.719			
	PAA 10	0.717			

*N* = 576. WS, weight stigma; BI, body image; PR, psychological resilience; PAA, physical activity avoidance; CR, composite reliability; AVE, average variance extracted.

**Table 4:** Heterotrait-Monotrait ratio (HTMT).

Constructs	Weight stigma	Psychological resilience	Body image	Physical activity avoidance
Weight stigma				
Psychological resilience	0.406			
Body image	0.384	0.513		
Physical activity avoidance	0.414	0.384	0.353	

**Table 5:** Discriminant validity (Fornell-Larcker criterion).

Constructs	Weight stigma	Psychological resilience	Body image	Physical activity avoidance
Weight stigma	**0.721**			
Psychological resilience	−0.374	**0.742**		
Body image	−0.345	0.460	**0.774**	
Physical activity avoidance	0.381	−0.351	−0.312	**0.719**

The square root of the AVE shown as the bolded diagonal values in the table.

### 4.3 Descriptive statistics and correlation analysis

The descriptive statistics, such as the mean (M), standard deviation (SD), skewness (SK), and kurtosis (Kur), are displayed in Table 6 together with the correlations between the primary variables to evaluate their distribution and central tendency. The data roughly followed a normal distribution and met the normality assumption for further regression analyses because all skewness and kurtosis values fell within the permissible range of ±2 for skewness and ±7 for kurtosis (George and Mallery, 1999).

**Table 6:** Correlation analysis of variables.

Constructs	M ± SD	SK	Kur	1	2	3	4
Weight stigma	3.024 ± 0.812	0.061	−0.337	1			
Body image	4.153 ± 1.444	0.527	0.445	−0.343***	1		
Psychological resilience	2.969 ± 0.841	0.034	−0.342	−0.371***	0.455***	1	
Physical activity avoidance	3.023 ± 0.803	−0.009	−0.470	0.375***	−0.312***	−0.347***	1

*N* = 576. *M*, mean; SD, standard deviation; SK, skewness; Kur, kurtosis; 1, weight stigma; 2, body image; 3, psychological resilience; 4, physical activity avoidance. Path significance: ***p < 0.001, the same below.

### 4.4 CMB

Using Harman’s single-factor test, we looked at CMB. The first component explained 28.825% of the variation, which is less than the 40% requirement (Podsakoff et al., 2003).

To provide a more rigorous assessment, we performed an unmeasured latent method construct (ULMC) analysis in AMOS 26.0. The baseline four-factor model showed good fit: χ^2^/df = 1.171, RMSEA = 0.017, CFI = 0.988. After adding a common method factor, the ULMC model also fit well: χ^2^/df = 1.153, RMSEA = 0.016, CFI = 0.989. The changes in fit indices were minimal (∆CFI = 0.001, ∆RMSEA = 0.001), well within recommended thresholds (Abd-El-Fattah, 2010). These findings suggest that common technique bias is not a significant issue in this investigation.

### 4.5 Multicollinearity diagnosis

We evaluated tolerance and VIF values to examine multicollinearity. Table 7 shows that VIF values ranged from 1.213 to 1.350, significantly below the typical threshold of 5, and all tolerance values were more than 0.1, suggesting that multicollinearity had little effect on the analysis (Hair et al., 2019).

**Table 7:** Multicollinearity diagnostics for the model.

Constructs	Tolerance	VIF
Weight stigma	0.824	1.213
Body image	0.758	1.319
Psychological resilience	0.741	1.350

*N* = 576. VIF, variance inflation factor.

### 4.6 Mediation effect analysis

All coefficients reported in the mediation analyses (Tables 8, 9) are standardized coefficients (*β*). Table 8 indicates that, after adjusting for gender, age, and grade, there was a negative correlation between weight stigma and psychological resilience and body image. Psychological resilience and body image were positively connected. Weight stigma was positively correlated with physical activity avoidance, while both body image and psychological resilience were negatively correlated with physical activity avoidance. These findings support H1.

**Table 8:** Regression analysis of mediator variables.

Result variable	Predictor variable	*β*	SE	*T*	Bootstrap 95% CI		*P*	*R* ^2^	*F*
					LLCI	ULCI			
Body image	Weight stigma	−0.344	0.039	−8.735	−0.421	−0.267	<0.001	0.121	13.025
Psychological resilience	Weight stigma	−0.245	0.039	−6.365	−0.320	−0.169	<0.001	0.261	28.712
	Body image	0.372	0.039	9.665	0.296	0.447	<0.001		
Physical activity avoidance	Weight stigma	0.255	0.041	6.172	0.174	0.336	<0.001	0.208	18.644
	Body image	−0.138	0.043	−3.214	−0.223	−0.054	<0.001		
	Psychological resilience	−0.191	0.044	−4.393	−0.276	−0.106	<0.001		

*N* = 576. *β*, standardized regression coefficient; SE, standard error; *t*, *t*-statistic; CI, confidence interval; LLCI, lower limit of confidence interval; ULCI, upper limit of confidence interval; R^2^, coefficient of determination; *F*, *F*-statistic. Bootstrap confidence intervals were based on 5,000 bootstrap samples. All *p*-values are two-tailed.

**Table 9:** Chain mediation of body image and resilience between weight stigma and activity avoidance.

Effect	Path	*β*	Bootstrap 95% CI		Percentage (%)
			LLCI	ULCI	
Total effect	Weight stigma →Physical activity avoidance	0.373	0.297	0.450	100.000
Direct effect	Weight stigma →Physical activity avoidance	0.255	0.174	0.336	68.365
Indirect effect	Weight stigma →Body image →Physical activity avoidance	0.048	0.015	0.084	12.869
	Weight stigma →Psychological resilience →Physical activity avoidance	0.046	0.024	0.074	12.332
	Weight stigma →Body image →Psychological resilience →Physical activity avoidance	0.024	0.012	0.039	6.434

*N* = 576. *β*, standardized regression coefficient; CI, confidence interval; LLCI, lower limit of confidence interval; ULCI, upper limit of confidence interval. Bootstrap confidence intervals and p-values were based on 5,000 bootstrap samples.

**Figure 2:**
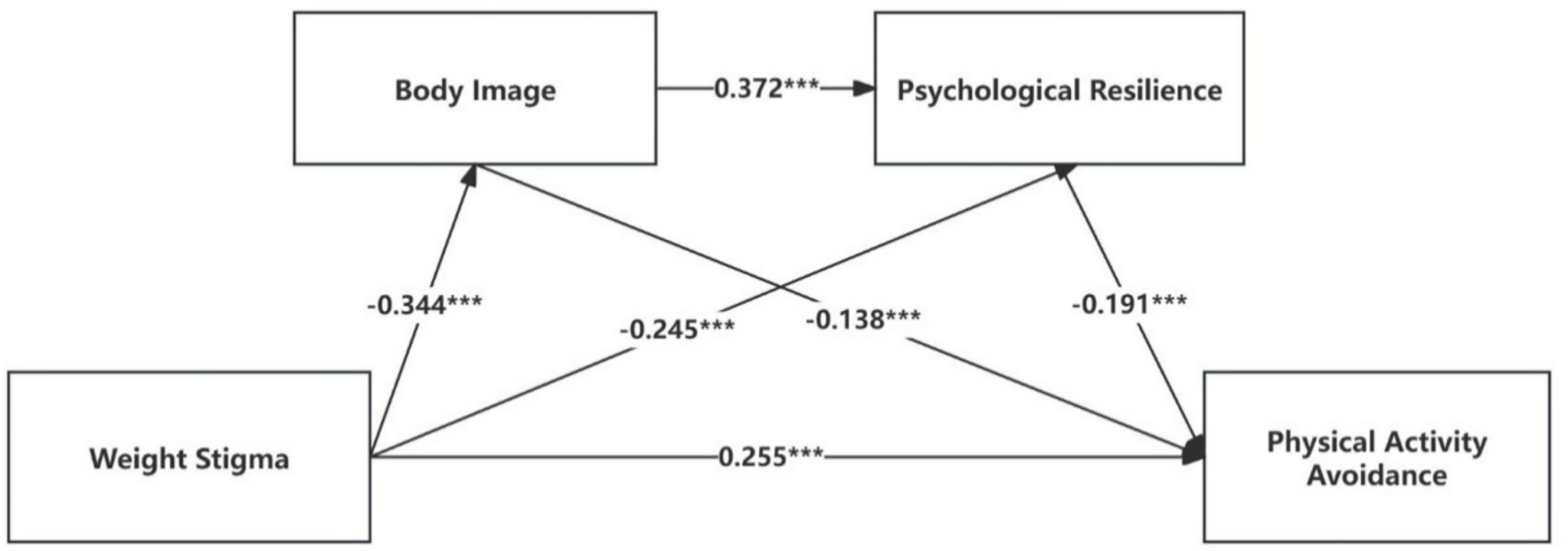
Mediation model. Numbers on the arrows represent standardized regression coefficients (*β* values). All displayed paths are statistically significant (***p < 0.001). Control variables were included but are not displayed.

The mediation analysis reveals a total effect of 0.373 and a direct effect of 0.255 in the association between physical activity avoidance and weight stigma, as indicated in Table 9 and Figure 2. The indirect effect associated with body image is 0.048, accounting for 12.869%, indicating a partial mediation by body image. The indirect effect associated with psychological resilience is 0.046, accounting for 12.332%, suggesting partial mediation by psychological resilience. The chain mediation effect through body image and psychological resilience is 0.024, representing 6.434% of the total effect. While this proportion appears modest, it holds practical significance in health contexts. Weight stigma is a complex societal factor not easily modified through individual-level interventions, and small effect sizes are common in public health research when distal predictors are involved (MacKinnon and Fairchild, 2009). This 6.43% reduction represents a meaningful target for health promotion programs. Thus, H2, H3, and H4 receive support.

## 5 Discussion

Guided by stress-coping theory, this study examined the relationship between college students’ physical activity avoidance and weight stigma, with a particular focus on the sequential mediation of psychological resilience and body image. The results generally support the proposed hypotheses. Weight stigma is not only directly associated with physical activity avoidance but also linked to behavior through its relationship with body image and psychological resilience. The following discussion further explores the findings and their psychological mechanisms.

First, this study found a strong positive correlation between college students’ physical activity avoidance and weight stigma, which is in line with new findings among Chinese university students (Huang et al., 2025; Q. Li et al., 2025). From the perspective of stress coping theory (Folkman, 1984), weight stigma functions as a chronic social stressor. Exercise settings often involve body exposure, peer comparisons, and being observed by others, conditions that may heighten the salience of this stressor. College students whose body weight does not conform to mainstream beauty standards may experience heightened levels of anticipated judgment or rejection in these situations. This perceived threat is associated with increased physical activity avoidance, as students with higher weight or lower body confidence may perceive exercise environments as unsupportive or unsafe (Saffari et al., 2025). In collectivist cultures, such as China, societal expectations for conformity to body image standards may be particularly pronounced due to the cultural emphasis on social harmony and group acceptance(Guidinger et al., 2021). This pressure to conform may heighten body image concerns and discourage physical activity participation. In contrast, in individualist cultures where personal autonomy is emphasized, college students may experience less pressure to conform to societal beauty standards, potentially resulting in different patterns of physical activity avoidance and coping mechanisms (Hong, 2011).

Second, among college students, the relationship between weight stigma and physical activity avoidance is significantly mediated by body image. This result is consistent with earlier studies showing that a higher perceived stigma around weight is linked to a worse body image, more body dissatisfaction, and more sensitivity to body exposure (Dever et al., 2025; Greenleaf and Rodriguez, 2021). Within the stress coping framework, body image can be understood as a cognitive appraisal process that shapes how college students interpret and respond to stigma-related stress. In cultural contexts that strongly emphasize social conformity, such as China, this appraisal process may be particularly sensitive to perceived deviations from prevailing beauty standards. College students may concentrate on perceived imperfections when weight stigma is linked to unfavorable body impressions, which increases feelings of shame and fear in workout environments (Borgatti et al., 2025). These results imply that body image is a significant psychological pathway that connects weight stigma to behavioral consequences, and that enhancing body image may lessen its detrimental correlation with avoiding physical activity.

Third, the association between weight stigma and college students’ physical activity avoidance was found to be significantly mediated by psychological resilience. College students reporting higher weight stigma tended to report lower psychological resilience, which in turn was associated with greater physical activity avoidance. This result is consistent with earlier studies showing that stigma around weight is frequently associated with lower resilience and more avoidance behaviors (Koreshe et al., 2023; Zhang et al., 2024). From the standpoint of stress coping, psychological resilience serves as a crucial individual resource that helps people efficiently handle stress associated with stigma. In the Chinese cultural context, where social evaluation and group harmony are highly valued, the anticipated judgment in exercise settings may pose a particularly salient threat (Guo et al., 2024). College students with lower resilience may struggle not only with internal emotional discomfort but also with concerns about disrupting social harmony or losing face (mianzi) in group exercise contexts. This dual burden, managing both internal distress and external social expectations, may further amplify avoidance tendencies, highlighting the importance of resilience building interventions that are culturally attuned.

Lastly, the association between weight stigma and physical activity avoidance is mediated sequentially by psychological resilience and body image. College students may find it more difficult to manage stress during workouts if they have fewer psychological resources, such as resilience, because weight stigma is negatively linked to body image. Long-term negative self-evaluation and self-denial may further diminish resilience (Shi et al., 2025), which is associated with increased avoidance of physical activity. Despite making up only 6.43% of the overall effect, the chain mediation effect is practically significant. In health behavior research, even small indirect effects can have meaningful public health implications when aggregated across populations (MacKinnon and Fairchild, 2009). The 6.43% proportion indicates that an intervention successfully enhancing body image and psychological resilience could potentially mitigate the total impact of weight stigma on physical activity avoidance by this magnitude. Given that weight stigma is a pervasive societal issue not easily amenable to individual-level change, targeting these malleable psychological mediators represents a pragmatic and potentially cost-effective approach. This modest effect at the individual level could translate into a meaningful reduction in health disparities when applied to the large population of college students who experience weight stigma.

### 5.1 Implications and limitations

#### 5.1.1 Theoretical implications

This study, based on the stress-coping theory, developed and tested a chain mediation model involving weight stigma, body image, psychological resilience, and physical activity avoidance, contributing to theoretical development.

First, the study defines weight stigma as a persistent social stressor and physical activity avoidance as a coping strategy in threatening exercise situations. This conceptualization meaningfully extends stress-coping theory by introducing weight stigma as a stressor in the context of health behaviors. By incorporating both into the same framework, it provides insights into how weight-related experiences affect health behaviors through stress appraisal and coping choices. Rather than reiterating the findings, this study emphasizes the “stress aspect” of weight stigma, offering a fresh perspective that links coping with weight-related stress to physical activity avoidance. This extension of stress-coping theory offers a deeper understanding of how external social stressors like weight stigma shape individuals’ coping strategies and health behaviors.

Second, by examining body image and psychological resilience as mediators, this study outlines a continuous path from external evaluations to internal experiences and behavior. This contributes to stress-coping theory by examining how weight stigma is associated with coping responses through its relationship with body image and psychological resilience. Psychological resilience shows a person’s capacity to handle stress, whereas body image shows how people feel and see their bodies. Together, these factors help clarify how weight stigma relates to physical activity avoidance. This broadens the application of stress-coping theory to weight-related experiences and explores how stigma affects psychological resources and behaviors.

Third, this study broadens research on weight-related psychological distress by examining physical activity avoidance in college students within the context of weight stigma. Unlike studies focusing mainly on emotional issues like depression, this study offers a broader view by showing how stigma limits health behaviors like physical activity. It integrates body image and resilience into the stress-coping framework, offering a comprehensive model that addresses both mental and behavioral health.

#### 5.1.2 Practical implications

At the individual level, this study shows that declines in body image and low psychological resilience are closely related to physical activity avoidance. This suggests that exercise interventions for college students should focus not only on physical fitness and skills but also on body awareness and stress responses. Through body acceptance training, improving body awareness, and learning effective emotional regulation strategies, students can reduce sensitivity to body exposure and strengthen their psychological resilience in exercise situations. Encouraging positive exercise experiences can also enhance self-efficacy and confidence in maintaining physical activity.

At the institutional level, the study highlights the importance of the campus exercise environment and teacher-student interactions. Universities should move beyond traditional competitive courses and create more inclusive environments that reduce comparison pressures. For example, offering a variety of exercise options, providing low-exposure or non-competitive classes, and changing evaluation methods can help reduce students’ concerns about body judgment. Physical education teachers should also be more sensitive to weight stigma and foster a supportive environment with encouraging feedback and respect for differences, increasing students’ sense of safety and willingness to participate in physical activities.

At the societal level, the connection between weight stigma and exercise avoidance underscores the need for caution in health promotion efforts. Public health campaigns should emphasize diverse body types and promote exercise values centered on function, health, and experience, rather than reinforcing a single body standard. Creating a more inclusive social and cultural environment at the macro level can reduce weight stigma, offering a supportive psychological context for college students to engage in physical activity.

### 5.2 Limitations and future research directions

Although there are still a number of limitations, this study offers preliminary evidence of the connection between psychological resilience, body image, weight stigma, and avoidance of physical activity among college students. This work can be expanded upon in the following ways by future research: First, self-report questionnaires were the main tool employed in this study. While these effectively capture participants’ perceptions and experiences, they may be influenced by biases such as social desirability and self-presentation. Future studies could use multi-source data, such as peer or teacher evaluations, exercise logs, structured classroom observations, and data from wearable devices, to provide more objective and comprehensive measurements of weight-related experiences and physical activity, improving reliability and ecological validity. Second, this study employed a cross-sectional methodology, which finds correlations between variables but does not establish causal linkages or monitor changes over time. A longitudinal method could be used in future research to examine how these factors interact and change over time. This would help identify how these characteristics evolve at different stages. Additionally, intervention studies might be created to evaluate the efficacy of resilience training, supportive exercise environments, or programs to enhance body image. These studies would assess how these factors reduce physical activity avoidance and provide empirical evidence for the causal pathways in the theoretical model, helping to apply research findings in educational practice. In addition, future studies should consider potential omitted variables such as self-esteem and social anxiety, which may also affect the relationship between weight stigma and physical activity avoidance. Including these factors in future research could offer a more comprehensive understanding of the mechanisms at play. Lastly, the sample used in this study was Chinese, and future research should take cultural specificity into account when interpreting the results. Examining these variables in various cultural contexts will assist ascertain whether the findings are applicable to other groups, as the relationship between weight stigma and physical activity avoidance may vary throughout cultures. Additionally, testing alternative models using larger samples and more diverse indicators could provide deeper insights into the psychological constructs under investigation. For instance, investigating how social support or other coping mechanisms influence the association between physical activity avoidance and weight stigma may help us better understand the mechanisms behind healthy behaviors and resilience in the face of stigma.

## 6 Conclusion

This study, which was based on stress-coping theory, examined the relationship between college students’ weight stigma and physical activity avoidance, emphasizing the mediating functions of psychological resilience and body image. The findings show that physical activity avoidance and weight stigma are significantly positively correlated, with resilience and body image acting as mediating factors in this relationship. Theoretically, this study expands the use of stress-coping theory in the field of weight management by highlighting the relevance of cognitive appraisal and psychological resources. Practically, it offers perspectives that may inform strategies to address physical activity avoidance among college students by considering both environmental and psychological conditions.

## Data Availability

The datasets presented in this study can be found in online repositories. The names of the repository/repositories and accession number(s) can be found in the article/supplementary material.
